# BODY DISSATISFACTION, PHYSICAL ACTIVITY, AND SEDENTARY BEHAVIOR IN FEMALE ADOLESCENTS

**DOI:** 10.1590/1984-0462/;2018;36;4;00005

**Published:** 2018

**Authors:** Valter Paulo Neves Miranda, Núbia Sousa de Morais, Eliane Rodrigues de Faria, Paulo Roberto dos Santos Amorim, João Carlos Bouzas Marins, Sylvia do Carmo Castro Franceschini, Paula Costa Teixeira, Silvia Eloiza Priore

**Affiliations:** a Universidade Federal de Viçosa, Viçosa, MG, Brasil.; b Universidade de São Paulo, São Paulo, SP, Brasil.

**Keywords:** Body image, Adolescent, Body composition, Physical activity, Sedentary lifestyle, Imagem corporal, Adolescente, Composição corporal, Atividade física, Estilo de vida sedentário

## Abstract

**Objective:**

To evaluate the association of body image with physical activity level, body composition, and sedentary behavior (SB) of female adolescents.

**Methods:**

Exploratory cross-sectional study conducted with 120 female adolescents aged between 14-19 years, from the city of Viçosa, Minas Gerais, Southeast Brazil. Body image was evaluated with a Body Silhouette Scale (BSS) and a Body Shape Questionnaire (BSQ). Weight, height, and waist circumference values were analyzed, as well as the waist-to-height ratio and body fat percentage. The physical activity level (PAL) was assessed by 24-hour Physical Activity Recall and SB by screen time, that is, time spent in front of a TV, playing video game, on the computer and using tablets, and, separately, the cell phone time.

**Results:**

Mean age was 16.5±1.5 years, and most adolescents were eutrophic (77.6%), sedentary/low PAL (84.2%), with high screen time (85.2%) and cell phone time (58.7%). Body dissatisfaction was stated in 40.6% of BSQ and 45.8% of BSS evaluations. Body distortion was identified in 52.9% of participants. All body composition measures, along with cell phone time and PAL, were associated with body dissatisfaction, the more active adolescents presenting higher levels of dissatisfaction.

**Conclusions:**

This study concluded that female adolescents with higher cell phone time also present higher body dissatisfaction, as well as the most physically active ones. All body composition measurements were associated with body dissatisfaction, mainly body mass index, waist circumference, and waist-to-height ratio.

## RESUMO

**Objetivo:**

Avaliar a associação da imagem corporal com o nível de atividade física, composição corporal e o comportamento sedentário de adolescentes do sexo feminino.

**Métodos:**

Estudo transversal e exploratório com 120 adolescentes do sexo feminino, de 14 a 19 anos, de Viçosa, MG. A imagem corporal foi avaliada por uma Escala de Silhuetas e pelo *Body Shape Questionnaire* (BSQ). O peso, a estatura e o perímetro da cintura foram aferidos, avaliando-se a relação cintura estatura e o percentual de gordura corporal. O nível de atividade física (NAF) foi avaliado pelo Recordatório de Atividade Física de 24h (R24h) e o comportamento sedentário, pelo tempo em frente à tela de uma TV, jogando videogame, ao computador e usando *tablets* e, separadamente, o tempo de tela ao celular.

**Resultados:**

A média de idade foi 16,5±1,5 anos, sendo a maioria das participantes eutróficas (77,6%), sedentárias/baixo NAF (84,2%), com tempo de tela (85,2%) e tempo de celular (58,7%) elevados. A insatisfação corporal manifestou-se em 40,6% pelo BSQ e em 45,8% pela Escala de Silhuetas. A distorção corporal manifestou-se em 52,9% das participantes. As medidas de composição corporal, juntamente com o tempo ao celular e o NAF, mostraram associação com a insatisfação corporal, sendo as adolescentes mais ativas as mais insatisfeitas.

**Conclusões:**

O estudo mostrou que as adolescentes com maior tempo ao celular tinham maior insatisfação corporal, assim como as fisicamente mais ativas. Todas as medidas de avaliação da composição corporal mostraram-se associadas ao nível de insatisfação, principalmente o índice de massa corporal, perímetro da cintura e relação cintura-estatura.

## INTRODUCTION

Adolescence is a period characterized by both physical and psychological transition, as well as behavioral changes, which can affect general health and well-being in adulthood.^1^ In this period, the sedentary lifestyle may result in increased food intake, leading to excessive caloric intake and body fat accumulation,^2^ one of the major causes of problems related to body image, especially among female adolescents.^3^

Body image is defined as an individual’s perception or feeling towards their own body size, shape, appearance, and silhouette. It’s a multidimensional construct supported by both attitudinal and perceptual dimensions. The attitudinal dimension of body image assesses cognitive, emotional, and behavioral aspects in addition to dissatisfaction with own body.^4^ ^,^ ^5^ Currently, a thin body is valued by the media, social environments, family, and friends.^6^ Overweight female adolescents can be considered part of a risk group for the development of body image disorders, since they internalize a strong desire to get skinnier. The aggravation of such disorders, associated with other factors, can trigger eating disorders (Table 1).^5^ ^,^ ^7^

**Table 1:** Association between independent variables and body dissatisfaction rating as per the Body Shape Questionnaire.

	BSQ (%)		p-value	PR	95%CI		p-value
	No dissatisfaction	Dissatisfaction					
BMI (n=101)							
Eutrophic/Low-weight	59 (58.4)	24 (23.8)	<0.001^b**^	1			<0.001
Overweight/obese	1 (1.0)	17 (16.0)		3.26	2.28	4.66	
BF% (n=97)							
Adequate	40 (41.2)	13 (13.4)	<0.001^a^	1			<0.001
Elevated	16 (16.5)	28 (28.9)		2.59	1.53	4.38	
WC (n=98)							
Adequate	58 (59.2)	32 (32.7)	0.006 ^b*^	1			<0.001
Elevated	1 (1.0)	7 (7.1)		2.46	1.67	3.61	
WHR (n=96)							
Adequate	56 (58.3)	29 (30.2)	<0.001^b**^	1			0.006
Elevated	1 (1.0)	10 (10.4)		2.66	1.87	3.78	
PAL (n=99)							
Active/very active	8 (8.1)	8 (8.1)	0.581	1			0.422
Sedentary/low PAL	50 (50.5)	33 (33.3)		0.79	0.45	1.38	
ST (2h) (n=99)							
Adequate	12 (12.1)	2 (2.0)	0.038 ^a*^	1			0.081
Elevated	46 (46.5)	39 (39.4)		3.21	0.86	11.90	
CT (2h) (n=98)							
Adequate	30 (30.6)	11 (11.2)	0.036 ^a*^	1			0.039
Elevated	29 (29.6)	28 (28.6)		1.83	1.03	3.24	

^a^Pearson’s chi-square test; ^*b*^ Fisher’s Exact test; *p<0.05; **p<0.001; BSQ: body shape questionnaire; PR: prevalence ratio; CI: confidence interval; BMI: body mass index; BF%: body fat percentage; WC: waist circumference; WHR: waist-to-height ratio; PAL: physical activity level; ST: screen time; CT: cell phone time.

Increase in usual physical activity levels and, consequently, decrease in sedentary behavior may boost energy consumption, thereby reducing overweight, raise body awareness, improve self-esteem, and reduce stress and anxiety, besides being an effective therapy for depression.^8^ However, the relation between physical activity level and body image remains unclear and should be investigated so that intervention programs that integrate obesity and eating disorders can be conceived. Thus, the aim of this study was to evaluate the association of body image with body composition, physical activity level, and sedentary behavior among female adolescents.

## METHOD

This is an exploratory, analytical, descriptive, cross-sectional study.^9^ A school with a large number of female adolescents in Viçosa, Minas Gerais, Southeast Brazil, was selected for convenience. The school was located within the facilities of Universidade Federal de Viçosa and the board of directors agreed with the research project. In 2014, 166 adolescents aged 14-19 years were enrolled in the school where the research was conducted, all of them being invited to participate. From the total, 148 agreed voluntarily to participate, but only 120 were considered eligible.

The power analysis was made in the statistical software OpenEpi^®^, version 3, (Bill & Melinda Gates Foundation, Atlanta, USA), taking into account the number of adolescents with or without body dissatisfaction as assessed by the Body Shape Questionnaire (BSQ).^10^ The post-hoc analysis showed a power of 87.5%, with continuity of correction factor.

The inclusion criteria were: female adolescents aged between 14 and 19 years that agreed voluntarily to participate in the study and had authorization of their caregivers when under 18 years old, without chronic or infectious diseases, not using contraceptive pills, not participating in any other study involving body composition assessment or nutritional status control.

The selected school was previously contacted and informed about the study. After the consent, the students were contacted for detailed explanation of the procedures to be performed. After having these documents delivered, social, demographic, and anthropometric data were collected, and all participants were assessed as to body image, physical activity level and sedentary behavior. All evaluations were performed in an appropriate room provided by the school, with the privacy necessary for data collection.

The weight was measured with an electronic digital scale Tanita BC-543^®^ (TMAB^®^, London, UK), and the height was measured by a portable stadiometer (Alturexata^®^, Belo Horizonte, Brazil). Body mass index (BMI) was calculated in the WHO AnthroPlus software and classified according to cutoffs proposed by De Onis et al.^11^

The body fat percentage (BF%), along with weight, was obtained using a Tanita BC-543 scale (TMAB^®^, London, United Kingdom) and classified according to cutoffs proposed by Williams et al:^12^ <25% eutrophic; ≥25% and <30% risk of overweight; ≥30% overweight.

Waist circumference (WC) was measured with a flexible, inelastic, 2-meter long tape (Cardiomed^®^, São Luis, MA, Brazil). The tape was placed at the midpoint between the lower border of the last rib and the iliac crest horizontally.^13^ The 90^th^ percentile of the population was adopted to classify measures, as recommended by the International Diabetes Federation.^14^

Waist-to-height ratio (WHR) was obtained by the ratio between waist circumference (cm) and the participant’s height (cm). The cutoff point adopted was 0.5, with variations suggested by Ashwell and Gibson.^15^

Body image was assessed using the Body Silhouette Scale developed by Kakeshita et al,^16^ which has been validated for Brazilian adolescents by Laus et al.^5^ It consists of 15 plastic card pictures numbered on the back. Each had a mean BMI value ranging from 12.5 to 47.5 kg/m², with differences between each level varying ±2.5 kg/m^2^.

The adolescents were individually evaluated and then shown card pictures, from the leanest to the thickest silhouette. First, the card representing their current silhouette (CS) was selected, followed by the one representing what they considered the ideal silhouette (IS). Body satisfaction was assessed by the difference between IS and CS. Body satisfaction was present when variation was between -1 and +1. When the difference was greater than +1, a desire for a bigger silhouette compared to participants’ current one was identified. If the difference was less than -1, the desire was towards a smaller silhouette.^5^

The BSQ assessed body dissatisfaction, more precisely regarding one’s overweight in the four weeks prior to data collection. The tool, validated for the Brazilian adolescent population by Conti et al,^10^ features 34 Likert-type items. Each question had six choices of answers, 1 being “never”; 6 “always”, with four other intermediate levels. As to the final score, less than 80 points means body satisfaction; 80-110 points, mild dissatisfaction; 110-140 points, moderate dissatisfaction, and ≥140 points indicates severe dissatisfaction.^10^

Physical activity level and sedentary behavior on weekdays were also evaluated. For physical activity, the 24-hour Physical Activity Recall (24h-PAR)^17^, adapted for adolescents by Bratteby et al,^17^ was used. This is a retrospective self-report instrument aimed at daily activities, with instructions and recommendations to identify and report the type of each activity performed throughout the day. The 24h-PAR was divided into 96 periods of 15 minutes each. The PAL was calculated based on the metabolic equivalent for adolescents and resting metabolic rate for female adolescents, as proposed by Schofield et al.^18^ In case any activity reported by the adolescents was not featured in the 24h-PAR, Ridley et al.’s compendium^19^ was used for Metabolic Equivalent (MET) classification. The answers to the physical activity questionnaire were categorized according to the cutoffs proposed by Brooks et al.^20^

Sedentary behavior was assessed based on the time spent in front a screen (screen time, ST), and the time spent on the cellular phone (cell phone time, CT) during the week, representing daily routine. ST was obtained by a questionnaire assessing the time spent in front of the television, computer, playing video games, and using tablets per day. CT was added and evaluated separately. Sedentary behavior was considered adequate when shorter or equal to two hours per day, and high when longer than two hours per day.^8^

Data were input to the Excel software by double typing. Statistical analyses were performed on Statistical Package for Social Sciences (SPSS) for Windows, version 20.0 (IBM Corporation^®^, New York, USA) and STATA version 13.0 (StataCorp LP^®^, Texas, United States). The Kolmogorov-Smirnov normality test showed that none of the variables had normal distribution; therefore, non-parametric tests were used. The level of rejection of null hypothesis was α=5%.

First, a descriptive analysis of variables was made. The total number of lost cases was displayed in tables for all variables assessed by both BSQ and BSS. Mann-Whitney and Kruskal-Wallis tests were used to compare two or more independent groups. The Bonferroni correction was used as post-hoc test for differences between groups. Associations were evaluated by the chi-square test (χ^2^), Fisher’s exact test, and Poisson regression in order to calculate gross and adjusted prevalence ratios (PR) for variables with p<0.20. The 95% confidence interval (95%CI) was also taken into consideration.

The Multiple Correspondence Analysis (MCA) was used to evaluate the correlation and variability of categorical variables. Categories distribution and internal correlation coefficient were investigated by Dimension Cronbach’s alpha Variance. By graphical representation, the associations could be interpreted according to the position of categories in a two-dimensional plan.^21^ Three or more dimensions were not used for they would considerably reduce Cronbach’s inertia and α values.^21^ Variables showing significant association upon the chi-square test were selected for the matching test.

This study was approved by the Ethics Committee on Research with Human Beings of Universidade Federal de Viçosa (UFV), Opinion number 700.976. This study complied with all the rules by the National Health Council, Resolution 466/12. All participants took part in the study only after having the Informed Consent Form and the Consent Agreement signed and handed by parents/caregivers and the participants, respectively.

## RESULTS

From 166 female adolescents in the school selected, 148 agreed voluntarily to take part in the sample and 120 female adolescents were eligible to do so. Mean age was 16.5±1.5 years. Body composition assessment identified 77.6% of the adolescents as normal-weight, and 20.7% as overweight and obese. The proportion of girls with adequate and high BF% was 53.3% and 46.7%, respectively. Upon WHR, 66.4% of results were considered adequate, 21.2% low, 10.6% high, and 1.9% very high.

Physical activity level evaluation sorted 84.2% of adolescents as “sedentary” or with “low physical activity level”, 14.2% as “active” and 1.5% as “very active”. Sedentary behavior was considered high in more than half of sample subjects. ST and CT exceeded 120 minutes in 85.2% and 58.7% of the girls, respectively.

Regarding body image assessment, 49 (40.83%) adolescents showed a degree of body dissatisfaction, with 22 (14.3%), 20 (19.8%) and 7 (6.9%) presenting mild, moderate, and severe dissatisfaction, respectively.

As per BSS, 65 (50.2%) girls were satisfied and 55 (49.8%) were dissatisfied with their body images. Among dissatisfied females, 29.4% wanted to have a leaner silhouette, while 20% wanted a thicker silhouette compared to their current status. A total of 64 (51.2%) subjects were related to body image distortion, with 32 (25.6%) seeing themselves as bigger (positive bias), and 32 (25.6%) as smaller than they actually were (negative bias).

**Figure 1:**
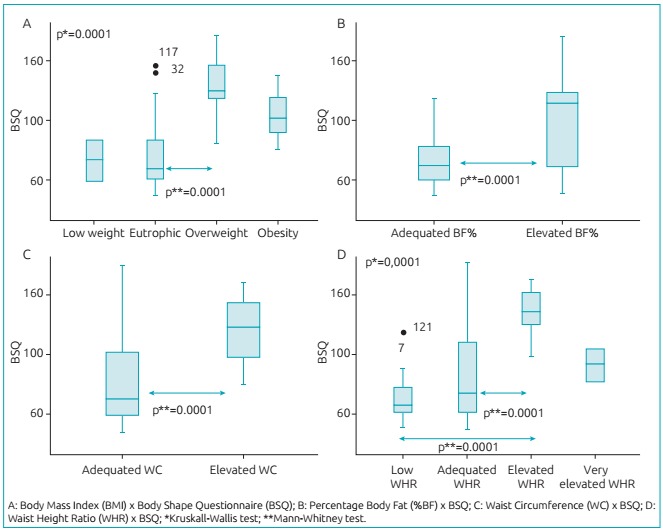
Evaluation of body image by Body Shape Questionnaire in relation to measures of body composition.

All groups with high BMI, BF%, WC, and WHR had significantly higher BSQ scores (Figure 1). There was an association between body dissatisfaction, assessed by the BSQ, and all body composition measurements. Adolescents with overweight/obesity were 3.26 times (95%CI 2.28-4.66) more prone to be considered dissatisfied with their body image when compared to eutrophic/low-weight adolescents. After being adjusted for age, BF%, WHR, PAL, and sedentary behavior, BMI was the only variable assessed by the questionnaire that was associated with body dissatisfaction.

Sedentary behavior and body dissatisfaction were found to be associated with both screen (χ^2^, p=0.038) and cell phone time (χ^2^, p=0.036). Adolescents with high CT values were 1.83 times (95%CI 1.03-3.24) more likely to present body dissatisfaction than girls with adequate CT, as per the crude Poisson regression.

A significant association was found between all body composition measures and dissatisfaction when assessed by the Body Silhouette Scale. WC and WHR had the highest prevalence ratios (PR): 2.17 (95%CI 1.57-2.98) and 2.05 (95%CI 1.46-2.89), respectively.

Physically active adolescents reported more dissatisfaction (PR=0.55, 95%CI 0.38-0.78) when compared to sedentary or low-activity subjects (Table 2).

**Table 2:** Association between independent variables and body dissatisfaction rating as per the Body Silhouette Scale.

Independent variables	Body Silhouette Scale Satisfaction		p-value	PR	95%CI		p-value
	Satisfied (%)	Dissatisfied (%)					
BMI (n=113)							
Eutrophic/low-weight	55 (48.7)	35 (31.0)	<0.001	1			<0.001
Overweight/obese	5 (4.4)	18 (15.9)		2.01	1.43	2.82	
BF% (n=117)							
Adequate	39 (33.3)	25 (21.4)	0.096	1	0.90	2.01	0.139
Elevated	25 (21.4)	28 (23.9)		1.35			
WC (n=104)							
Adequate	55 (52.9)	39 (37.5)	0.005	1	1.57	2.98	<0.001
Elevated	1 (1.0)	9 (8.7)		2.17			
WHR (n=103)							
Adequate	53 (51.5)	37 (35.1)	0.006	1	1.46	2.89	<0.001
Elevated	2 (1.9)	11 (10.7)		2.05			
PAL (n=120)							
Active/very active	5 (4.2)	14 (11.7)	0.011^a^	1	0.38	0.78	0.001
Sedentary/low PAL	60 (50.0)	41 (34.2)		0.55			
ST (2h) (n=117)							
Adequate	9 (7.7)	9 (7.7)	0.798	1	0.53	1.48	0.653
Elevated	55 (47.0)	44 (37.6)		0.88			
CT (2h) (n=116)							
Adequate	28 (24.1)	19 (16.4)	0.453	1	0.77	1.81	0.441
Elevated	36 (31.0)	33 (28.4)		1.18			

^a^Pearson’s chi-square test; ^*b*^ Fisher’s Exact test; *p<0.05; **p<0.001; BSQ: body shape questionnaire; PR: prevalence ratio; CI: confidence interval; BMI: body mass index; BF%: body fat percentage; WC: waist circumference; WHR: waist-to-height ratio; PAL: physical activity level; ST: screen time; CT: cell phone time.

After adjustment to BMI, WHR, WC, age, and CT, the physical activity level was the only variable whose prevalence ratio remained significant, suggesting higher dissatisfaction among physically active adolescents regardless of other confounding factors (PR=0.64, 95%CI 0.42-0.96). 

**Figure 2:**
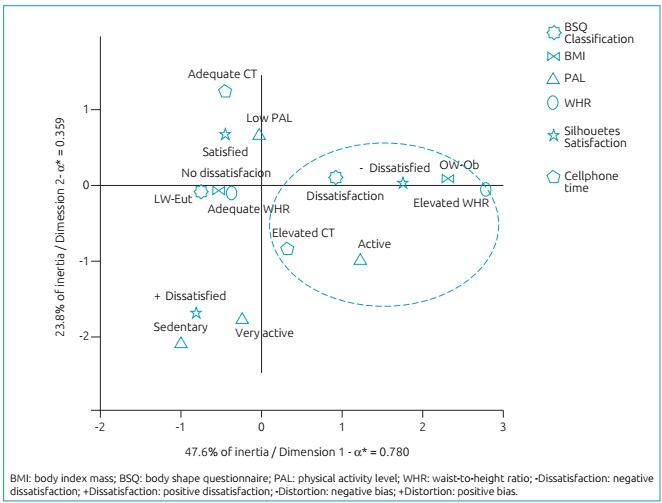
Multiple correspondence analysis between BMI, BSQ, Distortion, PAL, Silhouette scale, and WHR.

Figure 2 shows a geometrical representation of the MCA for variables’ categories in the factorial plan, with two dimensions. Dimension 1 explained 47.6% of data variability, with Cronbach’s α=0.780, which represents a satisfactory discriminatory power.^21^ Dimension 2 explained data variability with inertia value of 23.8% and Cronbach’s α=0.359, representing a moderate discriminatory power.^21^

## DISCUSSION

Body composition measurements, along with sedentary behavior and physical activity level, were shown associated with and/or to correspond to body dissatisfaction in female adolescents. Body dissatisfaction as evaluated by the BSQ was associated with overweight and obesity, and as evaluated by the BSS, with high waist circumference and waist-to-height ratio. More active female adolescents and those showing high CT also presented higher body dissatisfaction.

Almost half the sample showed dissatisfaction with weight and almost image. These results confirm the desire of adolescents to have a skinny body, without fat accumulation, as previously stated in the literature.^3^ ^,^ ^7^

The study by Sutter et al^6^ showed that female adolescents with overweight presenting high distortion of their physical appearance and severe body dissatisfaction were more vulnerable to physical, psychosocial, and physiological disorders. Adolescence is a stage of life in which one’s relationship with own body image can be more complex, especially for girls, due to the changes that occur in their bodies.^7^

Our results showed that overweight and fat accumulation in the central region of the body are important variables related to the feeling of depreciation of physical appearance. According to Moreno et al.^2^ and Saunders et al.,^22^ many adolescents spend most of their day time in a sedentary behavior, which is directly related to overweight and obesity. This study pointed more than half of adolescents with low PAL in addition to high ST and CT. These results are alarming, as the prevalence of overweight and obesity in children and adolescents is increasing in Brazil, reaching 30% of this population.^23^

Female adolescents with overweight and severe body dissatisfaction are at risk for dangerous eating habits and more likely to develop psychiatric disorders.^5^ ^,^ ^6^ ^,^ ^7^ ^,^ ^22^ Severe dissatisfaction with own body image is considered one of the main symptoms of eating disorders, which affects 0.1 to 5% of adolescents.^24^

PAL was associated with body dissatisfaction, and the adolescents who demonstrated a greater desire towards a different silhouette were the most physically active ones. Similarly, Rech et al.^25^ found high levels of body dissatisfaction in people with moderate and high PAL. Conversely, the PAL can be considered a form of overweight control and body awareness increase, factors that may influence positively one’s relationship with body image.^8^ ^,^ ^26^

Another result that is worth emphasizing was the high sedentary behavior adopted by more than half of the adolescents. CT was associated with body dissatisfaction. Importantly, few studies have assessed the relationship between body image and sedentary behavior while taking different screen devices into consideration. Other studies have pointed high sedentary behavior as the time spent in front of a TV and associated with low PAL and unhealthy eating habits.^2^ ^,^ ^8^ ^,^ ^22^ ^,^ ^27^

ST may be associated with the exposure to different online victimization situations, also known as “cyber bullying”. To Landoll et al.,^28^ cyber bullying is common and can be associated with the onset of self-esteem problems, depression, anxiety, and body image disorders. A population survey carried out in Brazil stated that 37% of children and adolescents aged 9-17 years had been discriminated on the Internet in the last 12 months, being exposed to hatred, intolerance, and violence speeches. In addition, 20% of interviewees reported experiencing cyber bullying before.^29^ According to the Brazilian Society of Pediatrics, adolescents should not be isolated in their rooms and exceed healthy sleep hours, besides being encouraged to practice at least one hour of physical activity daily.^30^

MCA was able to explained the 71.4% variability of data, meaning that body dissatisfaction and the desire of a leaner silhouette was related with overweight/obesity, increased waist circumference, high CT, and physical activity level. A combined analysis revealed an association between overweight and central obesity with negative feeling about body image, as also reported by Miranda et al,^21^ who found a correspondence between “severe body dissatisfaction, overweight/obesity and female gender”.

The cross-sectional design and the evaluation of only one day of PAL and sedentary behavior are limitations of this study, which does not allow a cause and effect analysis between such behaviors and body image. The small sample size can also be considered a limitation, but the study power was 87.49%, with continuity of the correction factor, thus ensuring analysis reliability. This was an exploratory study that pointed an association and correspondence between different components of body image and PAL, sedentary behavior, and different body composition measures.

Conclusion is that adolescents with high CT also had higher body dissatisfaction, as well as the most physically active ones. All body composition measurements were associated with body dissatisfaction, namely BMI, WC, and WHR. Further studies should be encouraged to assess the association between attitudinal and perceptual dimensions of body image and lifestyle factors, which may influence body composition and one’s assessment of own physical appearance. Therefore, educational and preventive measures may be taken to promote the healthy physiological and psychological development of this population.
